# Iohexol Degradation by Biogenic Palladium Nanoparticles Hosted in Anaerobic Granular Sludge

**DOI:** 10.3389/fmicb.2018.01980

**Published:** 2018-08-23

**Authors:** Xiangchun Quan, Xin Zhang, Yue Sun, Jinbo Zhao

**Affiliations:** Key Laboratory of Water and Sediment Sciences of Ministry of Education, State Key Laboratory of Water Environment Simulation, School of Environment, Beijing Normal University, Beijing, China

**Keywords:** iohexol, degradation, anaerobic granular sludge, palladium nanoparticle, hydrodehalogenation

## Abstract

To improve the degradation ability of anaerobic granular sludge (AGS) toward the iodinated contrast media (ICM) iohexol, biogenic nanoscale palladium (Pd) was formed in AGS via microbial reduction. The Pd hosted in AGS (Pd-AGS) was used for iohexol degradation. The effects of the electron donor, reaction medium, iodide ion fouling, and polymer embedding of the Pd-AGS on the reactivity were investigated. Our results showed the Pd-AGS increased the degradation rate of iohexol, with a conversion rate constant increased by 86.3-fold compared to the AGS control. Various organic compounds were investigated as electron donors to initiate the catalytic activity of Pd-AGS and the promotion achieved with the tested electron donors was in the following order: formate > lactate > ethanol > glucose > acetate. The Pd-AGS had high reactivity in deionized water at mild pH, and almost no reactivity under acidic (pH = 1.2) and alkaline (pH > 11) conditions. The presence of iodide ions in the medium inhibited the catalytic activity of Pd-AGS toward iohexol because of catalyst fouling. Embedding the Pd-AGS in alginate, chitosan, or polyvinyl alcohol (PVA) could prevent Pd loss but it also retarded the iohexol degradation rate. The Pd-AGS, as a combination of Pd catalyst and AGS, provides a novel strategy for iohexol degradation in polluted water and wastewater.

## Introduction

Iodinated contrast media (ICM) are a widely used class of pharmaceuticals for imaging in radiological and medical diagnoses (Perez and Barcelo, 2007). ICM compounds are often released to wastewater treatment systems via urine excreted from the human body. As ICM are generally metabolically stable, they can hardly be degraded in conventional biological wastewater treatment systems. Iohexol is a typical ICM, and is often used as diagnostic iodinated contrast agent in high doses in hospitals. In one standard Swiss university hospital, iohexol consumption reached 725 g/day (Weissbrodt et al., 2009) and half this was discharged in wastewater. The concentration of iohexol detected in surface water bodies and wastewater effluent is in a range from ng/L to μg/L, which will pose a high risk to the environmental biota (Chèvre, 2014).

Many methods have been investigated to enhance ICM degradation, such as ultraviolet-based advanced oxidation (Giannakis et al., 2017), sulfate-mediated electrooxidation (Radjenovic and Petrovic, 2016), and biofilm technology (Escola Casas and Bester, 2015). Recent studies have found that biogenic palladium (Bio-Pd) can be used for oxidative and reductive removal of pharmaceuticals, biocides, and ICM (Forrez et al., 2011). Palladium nanoparticles (PdNPs) are effective in catalyzing a variety of reactions, such as hydrodehalogenation (HDC) (Hennebel et al., 2009b), denitrification (Liou et al., 2012), and azo dye reduction (Johnson et al., 2013). PdNPs can reduce halogenated compounds through HDC process after being charged with molecular hydrogen in the presence of a hydrogen donor (Hennebel et al., 2009b).

Palladium nanoparticles are often fabricated using chemical methods with lots of chemicals under harsh conditions. Preparation of PdNPs through microbial reduction is regarded as a promising and environmentally friendly method as it uses less toxic chemicals and no stabilizers or carriers (Shen et al., 2003). Unlike nanoparticles prepared using chemical methods, which are generally not stable, easily aggregate with activated sludge and influence sludge microbial community (Zhou et al., 2015; Zhang et al., 2016), the PdNPs formed by the microbes are in good dispersion and more stable (Hennebel et al., 2011). Biogenic Pd can be produced by some pure strains (Hennebel et al., 2011; De Corte et al., 2012) and also by some mixed microbial cultures such as anaerobic granular sludge (AGS) (Quan et al., 2015). We previously found that AGS could form Bio-Pd in its structure because of the existence of Pd (II) reducing bacteria (Quan et al., 2015). AGS is a special form of microbial aggregates with a three-dimensional and heterogeneous structure comprising a complex microbial culture (Hulshoff Pol et al., 2004). AGS based anaerobic processes are widely used for high strength and recalcitrant organic wastewater treatment (Lim and Kim, 2014). Hosting of Bio-Pd in AGS (Pd-AGS) provides a novel form of AGS, which combines the function of microbial metabolism with chemical catalysis. We previously investigated azo dye reduction by Pd-AGS (Quan et al., 2015). However, application of the Pd-AGS for HDC of ICM has not been well investigated to date.

In this study, Bio-Pd was prepared using AGS as the Pd (II) reducer and immobilization carrier. The reaction activities of the Pd-AGS toward iohexol were evaluated with different electron donors, reaction mediums, and immobilization methods. This study provides detailed information on the application of Pd-AGS to degradation of ICM.

## Materials and Methods

### Chemicals and Materials

Iohexol (**Supplementary Figure S1**) was purchased from Sigma-Aldrich (purity >95%). The AGS for Bio-Pd production was obtained from a full-scale anaerobic reactor treating wastewater from beer production in Beijing, China. The AGS had a mean diameter of approximately 5 mm and good settling ability with a Sludge Volume Index of around 20. Unless otherwise stated, all the reagents used in this study were of analytical grade.

### Preparation of Pd-AGS

The Pd-AGS was prepared as described in our previous study (Quan et al., 2015). Briefly, AGS pellets were added to serum bottles to give a final biomass concentration of 2 g Volatile Suspended Solids (VSS)/L. Then, a certain volume of sodium formate stock solution was added (final concentration of 25 mM) to act as an electron donor. The remaining space in each bottle was filled with M9 medium. The serum bottles were subsequently flushed with nitrogen gas for 15 min to remove air and create an anaerobic environment. Then, they were spiked with a predefined volume of Na_2_PdCl_4_ stock solution (final concentration of 100 mg/L). After being tightly sealed with inert Viton stoppers, the bottles were incubated in a shaker (180 rpm) at 35 ± 1°C for 8 h to reduce Pd (II). The Pd-AGS in the reaction medium was subsequently collected by centrifugation at 3000 × *g* for 10 min, and then gently washed with distilled water three times in preparation for use in the iohexol reduction experiments. The PdNPs can be formed in the cell walls, periplasmic space, or cytoplasm of microbes of AGS (**Supplementary Figure S2**; Quan et al., 2015). The M9 medium contained the following compositions (g/L): 12.8 Na_2_HPO_4_⋅7H_2_O, 3 KH_2_PO_4_, 0.5 NaCl, 1.0 NH_4_Cl, 0.24 MgSO4, 0.011 CaCl_2_.

### Reactivity of Pd-AGS Toward Iohexol Under Different Conditions

Degradation of iohexol by the Pd-AGS was assessed in the presence of various electron donors (sodium formate, glucose, sodium acetate, ethanol, and sodium lactate). First, Pd-AGS or AGS control pellets (final AGS concentration of 2 g VSS/L) were added to serum bottles (50 mL total volume, 30 mL working volume). Then, the reaction medium and electron donor (each at 500 mg/L), and iohexol stock solution (final concentration of 20, 50, or 100 mg/L) were added. The bottles were flushed with N_2_ gas or hydrogen (for the hydrogen donor system) for 10 min to remove oxygen, and finally tightly sealed with stoppers and incubated on a shaker (180 rpm) at 35 ± 1°C. Samples were withdrawn after specific intervals and the concentration of iohexol remaining in each sample was measured.

To investigate the effects of the reaction medium on iohexol degradation by Pd-AGS, similar degradation experiments were conducted in deionized (DI) water (pH = 6.9), phosphate buffered saline (PBS, pH = 7.5), an acidic medium (pH = 1.2) spiked with H_2_SO_4_, or an alkaline medium containing Na_2_CO_3_ (pH = 11.2) or NaOH (pH = 11.7).

In addition, the effect of iodide ions on iohexol degradation by the Pd-AGS was investigated with various iodide ion concentrations. We used iodide ion concentrations of 0.073, 3.65, 7.3, and 36.5 mM, which corresponded to 1, 50, 100, and 500 times the theoretical iodide ion concentration released from 20 mg/L iohexol, respectively.

### Effects of Polymer Immobilization on the Reactivity of Pd-AGS

To prevent Bio-Pd loss from Pd-AGS, we attempted to embed the Pd-AGS in sodium alginate, chitosan, or polyvinyl alcohol (PVA) using the following procedure. First, the Pd-AGS was collected and gently washed with a PBS solution three times. Chitosan was dissolved in an acetic acid solution (2.5% v/v) to make a 5% (w/v) chitosan solution. Sodium alginate was dissolved in distilled water to make a 3% (w/v) alginate solution. A solution of PVA (10% w/v) was made by dissolving PVA in distilled water. The AGS or Pd-AGS (final AGS concentration of approximately 2 VSS g/L) was then added to the above gel solutions and mixed completely. The gel–biomass mixture was then dropped into a saturated boric acid solution containing 2% CaCl_2_ with a syringe to yield PVA or alginate gel beads, or dropped into glutaraldehyde (0.5%) solution containing 2% CaCl_2_ to yield chitosan gel beads. Crosslinking at 25 ± 1°C for 24 h generated gel beads with a diameter of 5–7 mm. These gel beads were then washed and used for iohexol degradation.

### Analytical Methods

The concentration of iohexol was analyzed using an ultra-performance liquid chromatography (Acquity System; Waters, Milford, MA, United States) with a 2996 photodiode array detector at a detection wavelength of 237 nm. The mobile phase was a mixture of methanol and water (7:93) (v/v) at a flow rate of 0.3 mL/min. Iodide ion analysis was performed by ion chromatography (ICS-1100, Dionex, United States) using 35 mM NaOH for elution. Total organic carbon (TOC) was measured using a TOC-V analyzer (Shimadzu Corporation, Japan).

## Results and Discussion

### Effects of Electron Donors on Iohexol Degradation by Pd-AGS

**FIGURE 1:**
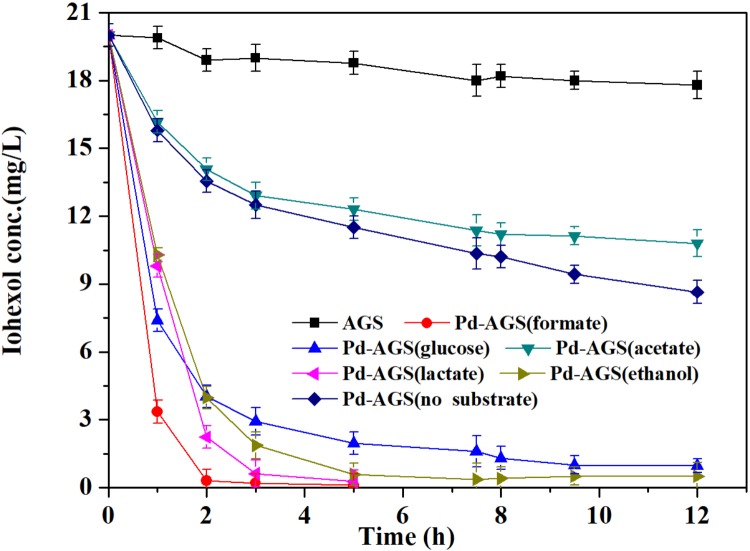
Effects of electron donors on iohexol degradation by Pd-AGS. Reaction conditions: initial iohexol concentration (*C*_0_) = 20 mg/L; sodium formate, glucose, sodium acetate, ethanol, or lactate (each at 500 mg/L) as the electron donor; 4 wt% Pd-AGS (2 g); 35 ± 1°C; 180 rpm; DI water; and pH = 6.9.

The Pd-based HDC process requires hydrogen and electron donors to initiate the catalyst. The effects of various electron donors (formate, lactate, ethanol, acetate, and glucose) on the reactivity of Pd-AGS toward iohexol were investigated (**Figure 1**). Only 11% of the iohexol was removed by the AGS control after 12 h in the presence of formate. With Pd-AGS, 51–100% of the iohexol was removed in the presence of the tested electron donors. These data indicate that combination of Bio-Pd with AGS greatly increased iohexol degradation. Except for acetate, all of the tested electron donors promoted iohexol degradation. The resulting data were well fitted to a pseudo-first order kinetics model based on the following equations:

(1) $$\frac{d[\text{IOX}]}{\mathrm{dt}}=-k_{\text{obs}}[\text{IOX}],$$

and

(2) $$\ln\left(\frac{{\left[\text{IOX}\right]}_{0}}{{\left[\text{IOX}\right]}_{\text{t}}}\right)=k_{\text{obs}}t,$$

where [IOX]_t_ is the concentration of iohexol (mg/L) at time *t*, [IOX]_0_ is the initial concentration of IOX (mg/L), and *K_obs_* is the observed first-order rate constant (min^−1^). Conversion rate constants were obtained with different electron donors (**Table 1**).

**Table 1:** The effects of various electron donors on the iohexol conversion rate (*k_obs_*).

Electron donor	AGS type	*K_obs_* (min^−1^)	*R*^2^
Formate	AGS	0.0004 ± 0.00005	0.793
Formate	Pd-AGS	0.0349 ± 0.0012	0.993
Lactate	Pd-AGS	0.0198 ± 0.0010	0.982
Glucose	Pd-AGS	0.0106 ± 0.00005	0.947
Ethanol	Pd-AGS	0.0135 ± 0.00006	0.996
Acetate	Pd-AGS	0.0026 ± 0.0001	0.954
Non	Pd-AGS	0.0026 ± 0.0001	0.954

The Pd-AGS had a higher *K_obs_* (0.0135–0.0349 min^−1^) than the AGS control (0.0004 min^−1^), with *K_obs_* increased by 32- to 86-fold. Compared to the experiment conducted with no electron donor, the *K_obs_* for the Pd-AGS increased by factors of 13.4, 7.6, 5.2, and 4.1 in the presence of formate, lactate, ethanol, and glucose, respectively. Iohexol was degraded to a certain extent even without the presence of an electron donor, possibly because some microbial lysis or digestion products released from the Pd-AGS could serve as electron donors to initiate the Pd activity. The promotion achieved with the tested electron donors was in the following order: formate > lactate > ethanol > glucose > acetate. We previously investigated azo dye reduction using Pd-AGS in the presence of various electron donors, and obtained similar results to the present study (Quan et al., 2015). After hydrogen, formate is reportedly an effective electron donor for use as a Pd-based catalyst, and it can be charged to surface Pd in the form of hydrogen hydride (Hennebel et al., 2009a). The Pd-AGS, as a combination of Pd catalyst and live microbial granule, may also utilize complex organic compounds as electron or hydrogen donors through biotransformation process. Fermentative bacteria in AGS may possess the ability to ferment glucose and generate volatile fatty acids and hydrogen, which may serve as an electron donor to initiate the Pd catalyst. Among the electron donors tested, acetate showed the lowest promotion to the Pd catalyst, possibly because it could not be effectively charged to Pd or transformed to hydrogen or other effective electron donors during fermentation (Logan et al., 2002).

**FIGURE 2:**
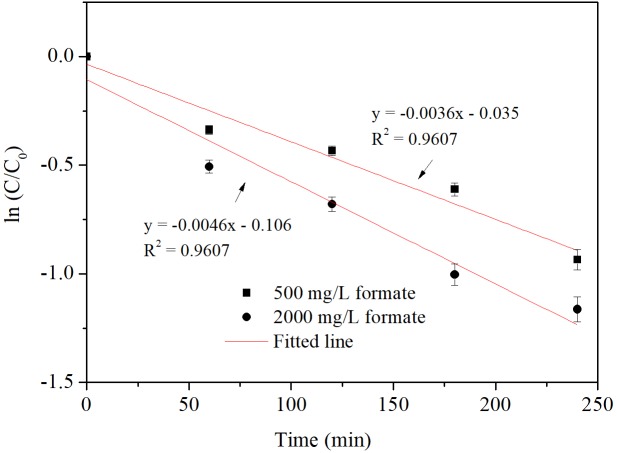
Reaction kinetics of iohexol by Pd-AGS in the presence of formate of different concentrations. Reaction conditions: initial iohexol concentration (*C*_0_) = 20 mg/L, sodium formate (500 or 2000 mg/L) as the electron donor, 4 wt% Pd-AGS (2 g), 35 ± 1°C, 180 rpm, DI water, and pH = 6.9.

Formate was most effective electron donor for iohexol conversion by Pd-AGS in this study. The effects of the formate concentration on the reactivity of Pd-AGS were further investigated (**Figure 2**). The iohexol conversion rate constant increased from 0.0036 to 0.0046 min^−1^ when the formate concentration was increased from 500 to 2000 mg/L. Therefore, sufficient formate is needed to obtain a high iohexol conversion rate.

### HDC Effects of Iohexol by Pd-AGS

**FIGURE 3:**
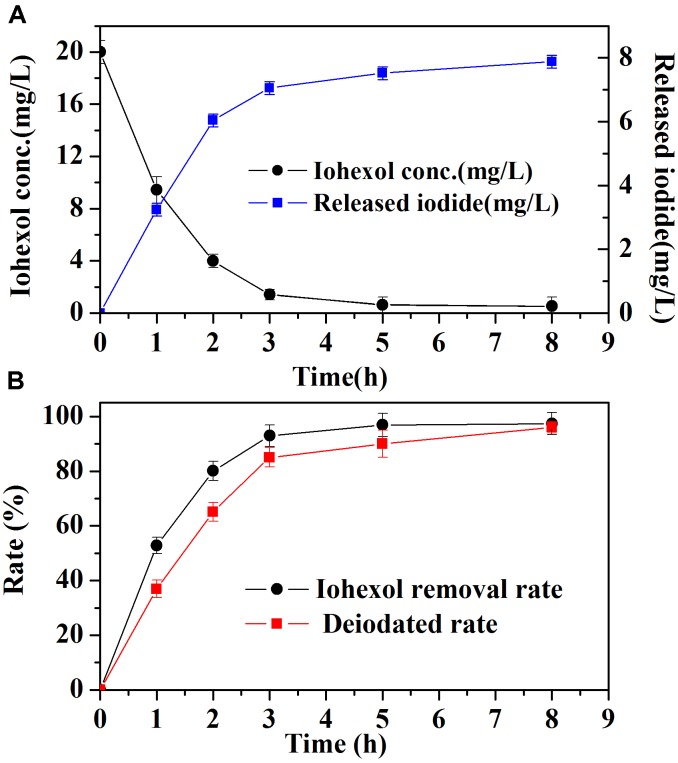
The removal of iohexol **(A)** and iodide ion **(B)** release during iohexol conversion by Pd-AGS. Reaction conditions: initial iohexol concentration (*C*_0_) = 20 mg/L, 500 mg/L sodium formate as the electron donor, 4 wt% Pd-AGS (2 g), 35 ± 1°C, 180 rpm, DI water, and pH = 6.9.

Iodide ion release during iohexol degradation by Pd-AGS was investigated further. Iohexol was degraded rapidly at the beginning of the reaction, and this was accompanied by rapid iodide ion release (**Figure 3A**). During the first 3 h, the iodide ion release rate (85%) was slightly lower than the iohexol removal rate (93%), possibly because the initial adsorption of iohexol on the surface of Pd-AGS may contribute partially to its removal. After 8 h, both the iohexol removal and iodide ion release rate reached 95% (**Figure 3B**). The C-I bond is weaker than a C-Br, C-Cl, or C-F bond, which may be a reason for the high release rate of iodide ions from iohexol degraded by Pd-AGS (Urbano and Marinas, 2001; Mackenzie et al., 2006). For the AGS control, only 11% of iohexol was removed (**Figure 1**), suggesting adsorption and biodegradation by the AGS were not the primary reasons for iohexol removal. The removal of TOC was also measured during the iohexol degradation by Pd-AGS (data not shown), and less than 15% was removed at the end of reaction. Based on these data, it could be deduced that deiodination of iohexol through catalytic reduction by Pd catalyst was the main mechanism for iohexol removal by the Pd-AGS.

**FIGURE 4:**
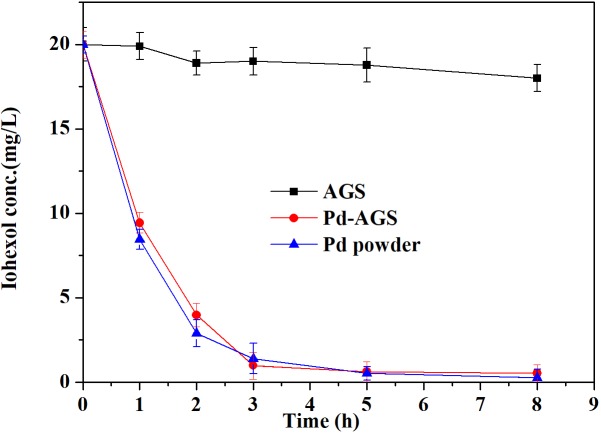
Comparative degradation of iohexol by Pd powder and Pd-AGS. Reaction conditions: initial iohexol concentration (*C*_0_) = 20 mg/L, 500 mg/L sodium formate as the electron donor, 4 wt% Pd-AGS (2 g), 35 ± 1°C, 180 rpm, DI water, and pH = 6.9.

The degradation of iohexol by the Pd-AGS, the AGS control and the corresponding same loading amounts of Pd power in the presence of electron donor formate were compared and the results were presented in **Figure 4**. After 8 h of reaction, 10% of the iohexol was removed using the AGS control. By contrast, both Pd-AGS and Pd powder showed rapid removal of iohexol and approximately 80% removal after 3 h of reaction. The Pd-AGS showed a similar degradation rate to the corresponding free Pd powder, which shows PdNPs immobilized in AGS has similar catalytic activity to free Pd powder. PdNPs is often immobilized on various supports to prevent catalyst loss and recover the catalyst easily during application (Creamer et al., 2007; Bennett et al., 2013). Our use of AGS is a novel strategy for PdNPs fabrication and *in situ* immobilization, and this technique effectively retains the catalytic activity of Pd.

### Reaction Kinetics of Iohexol Degradation by Pd-AGS at Different Concentrations

**FIGURE 5:**
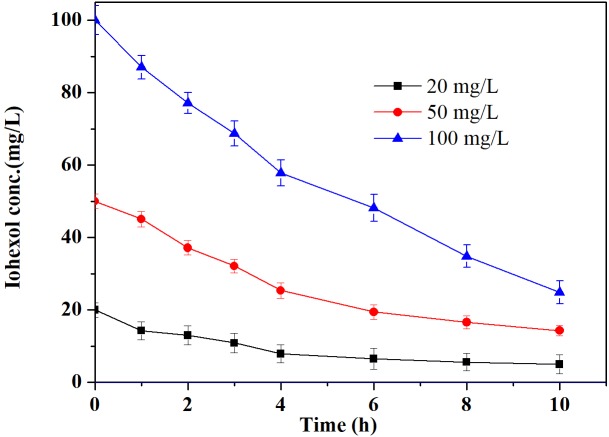
Effects of the initial concentration of iohexol on degradation by Pd-AGS. Reaction conditions: initial iohexol concentration (*C*_0_) = 20, 50, or 100 mg/L; 500 mg/L sodium formate as the electron donor; 4 wt% Pd-AGS (2 g); 35 ± 1°C; 180 rpm; DI water; and pH = 6.9.

The reaction kinetics of the Pd-AGS degradation of iohexol at various concentrations was investigated further (**Figure 5**). The initial iohexol degradation rates were 2.771, 6.231, and 9.587 mg/(L⋅h) with iohexol concentrations of 20, 50, and 100 mg/L, respectively. Increasing the initial concentration of iohexol led to an increased initial degradation rate, indicating a positive correlation between the adsorption of iohexol on the Pd-AGS and the Pd-AGS catalytic activity. This dependence was clarified by fitting the experimental data to the Langmuir–Hinshelwood model (de Pedro et al., 2010), which is given by the following equations:

(3) $${\text{r}}_{0}=k\theta_{\text{s}}=k\frac{{\mathrm{bC}}_{0}}{1+{\mathrm{bC}}_{0}}$$

(4) $$\frac{1}{{\text{r}}_{0}}=\frac{1}{{\mathrm{kbC}}_{0}}+\frac{1}{k}$$

where *r*_0_ is the initial degradation rate at an iohexol concentration of *C*_0_, 𝜃_S_ is the coverage of iohexol adsorbed on the catalyst surface, *k* is the reaction rate constant, and *b* is the equilibrium constant for iohexol adsorption. A plot of 1/*r*_0_ versus 1/*C*_0_ showed a good linear relationship with a correlation coefficient (*R*^2^) of 0.99. This result indicated that the catalytic degradation of iohexol by the Pd-AGS followed the Langmuir–Hinshelwood model and the conversion of adsorbed iohexol on the catalyst surface was the rate controlling step.

### Effects of the Reaction Medium on Iohexol Degradation by Pd-AGS

**FIGURE 6:**
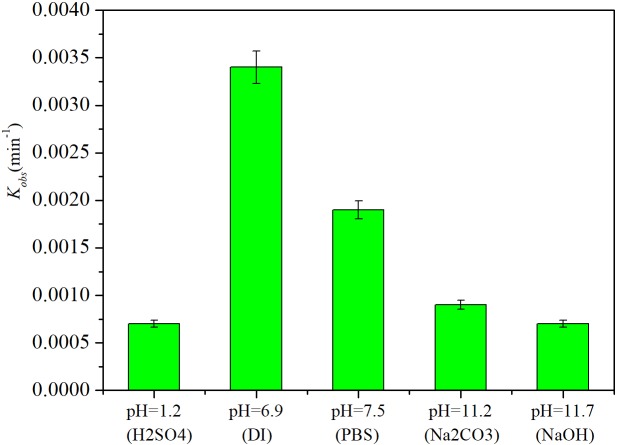
Effects of the reaction medium and pH on iohexol degradation by Pd-AGS. Reaction conditions: iohexol initial concentration (*C*_0_) = 20 mg/L; 500 mg/L sodium formate as the electron donor; 4 wt% Pd-AGS (2 g); DI water (pH = 6.9), PBS (pH = 7.5), alkaline medium adjusted using NaOH (pH = 11.7), alkaline medium adjusted using Na_2_CO_3_ (pH = 11.2), or acidic medium (pH = 1.2) adjusted using H_2_SO_4_; 35 ± 1°C; and 180 rpm.

In the HDC process mediated by a Pd catalyst, the pH and halogenated ions may influence the reaction (Creamer et al., 2007; Yuan and Keane, 2007; Xia et al., 2009). The degradation of iohexol was investigated in different reaction media and under different pH conditions (**Figure 6**). The highest iohexol degradation rate was obtained in DI water (pH = 6.9), with a kinetic rate constant of 0.0034 ± 0.0002 min^−1^. In PBS (pH = 7.5), the degradation rate of iohexol was slightly retarded compared with that in DI water and the rate constant decreased to 0.0019 ± 0.0001 min^−1^. This retardation could be caused by Pd poisoning by phosphate ions (Felis et al., 1999). When the degradation was conducted in an alkaline medium containing NaOH (pH = 11.7) or Na_2_CO_3_ (pH = 11.2), the degradation of iohexol was significantly inhibited (*p* < 0.05) and the rate constant decreased to 0.0009 ± 0.00005 and 0.0007 ± 0.00003 min^−1^, respectively. This could occur because the presence of OH^−^ could inhibit the reactivity of the Pd catalyst by blocking Pd^n+^ active sites (de Pedro et al., 2011). Others have also studied the effects of base addition on HDC using Pd catalysts, but complicated results were obtained (Yuan and Keane, 2007; Xia et al., 2009). One study found that addition of a base increased the HDC reactivity of Pd because the base could neutralize any hydrogen halides produced and make the catalyst free from Cl^−^ (Creamer et al., 2007). The presence of CO_3_^2-^ or HCO_3_^−^ reportedly increases the Pd/C catalyst reactivity toward 4-chlorophenol (Xia et al., 2009). However, the effects of base addition on the HDC reaction were also pH dependent. de Pedro et al. (2011) found that increasing the pH within the range 8.5–9.5 increased the 4-chlorophenol conversion rate, and pH increases beyond that range resulted in more OH^−^ that inhibited the reaction because of coverage of the Pd^n+^ active sites. Yuan and Keane (2007) studied HDC of 2,4-dichlorophenol in an aqueous solution with a Pd/Al_2_O_3_ catalyst, and found high catalytic activity was achieved at pH 7–8. Hennebel et al. (2010) put a Pd catalyst into a membrane reactor to remove diatrizoate and obtained high removal in mild and alkaline media. In this study, iohexol degradation was also significantly retarded in an acidic medium (pH = 1.2), possibly because of catalyst loss and catalytic activity inhibition under highly acidic conditions.

### Effects of Iodide Ions on HDC of Iohexol by Pd-AGS

**FIGURE 7:**
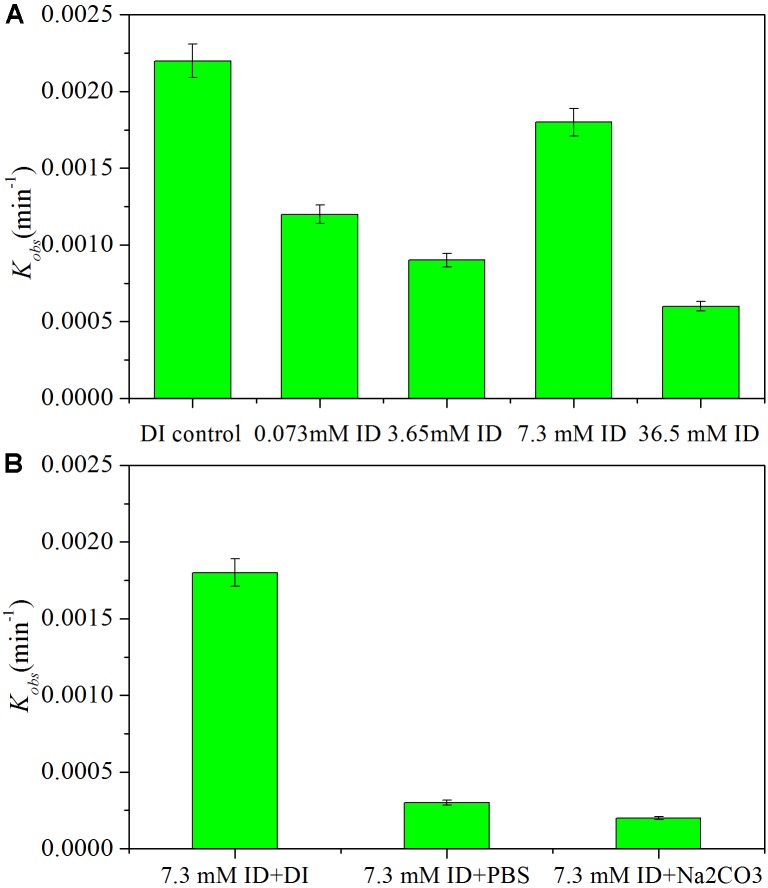
Effects of iodide ion (ID) on iohexol degradation in deionized (DI) water **(A)** or in a PBS or Na_2_CO_3_ buffered medium **(B)**. Reaction conditions: iohexol initial concentration (*C*_0_) = 20 mg/L; 500 mg/L sodium formate as the electron donor; 4 wt% Pd-AGS (1.2 g/L); DI water (pH = 6.9), PBS (pH = 7.5), or Na_2_CO_3_ buffered medium (pH = 11.2); iodide ion concentration 0, 0.073, 3.65, 7.3, or 36.5 mM; 35 ± 1°C; and 180 rpm.

Released halide ions may influence the HDC process with a Pd catalyst. Degradation of iohexol by Pd-AGS was investigated in the presence of iodide ions at various concentrations (0.073, 3.65, 7.30, and 36.5 mM iodide ion) (**Figure 7**). The results showed that the presence of iodide ions retarded iohexol degradation significantly (*p* < 0.05), and the kinetic rate constant decreased by 45–73% compared with that in the DI water (**Figure 7A**). Iodide ions have a strong affinity to the surface of the Pd catalyst and can block its active sites, which may contribute to inhibition of the HDC reaction (Mackenzie et al., 2006). When the degradation was conducted in PBS or Na_2_CO_3_ buffered medium containing iodide ions, iohexol degradation was inhibited more strongly compared with that in DI water (**Figure 7B**). In most cases, the iodide concentration in water or wastewater is much lower than the concentration tested in this study and the iodide ion fouled Pd catalyst can be restored through DI water washing.

### Effects of Polymer Immobilization on Pd-AGS Reactivity

**FIGURE 8:**
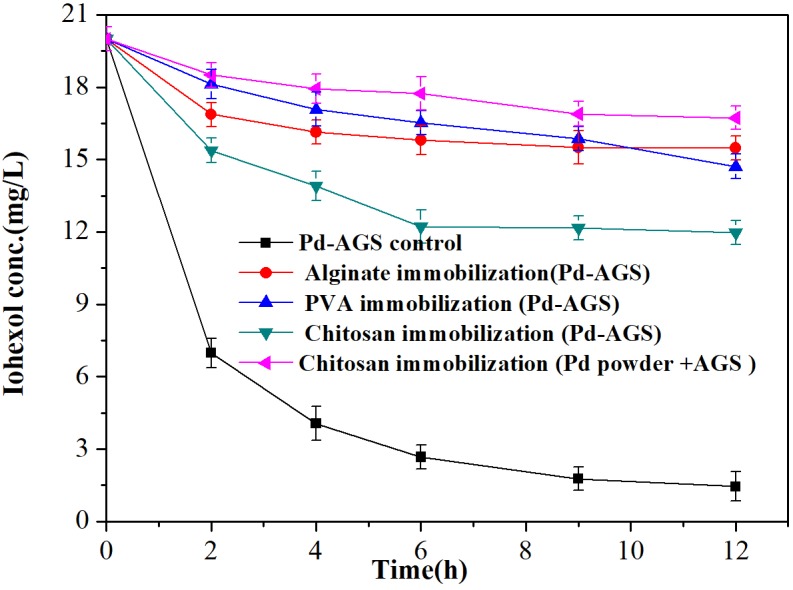
Effects of polymer immobilization of Pd-AGS on the degradation of iohexol. Reaction conditions: iohexol initial concentration (*C*_0_) = 20 mg/L, 500 mg/L sodium formate as the electron donor, 2 wt% Pd-AGS (2 g/L) embedded in a polymer, DI water, 35 ± 1°C, and 180 rpm.

As some microbes in AGS may suffer toxicity from Pd (II) during reduction process and therefore detach from AGS, we investigated embedding the Pd-AGS in polymer alginate, chitosan, or PVA to prevent the biomass and catalyst loss. The effects of immobilization on the Pd-AGS reactivity were investigated (**Figure 8**). Immobilization using chitosan, alginate, and PVA decreased the catalytic activity, and kinetic rate constant decreased to 18.9, 9.3, and 8.4%, respectively, of the original Pd-AGS. Immobilization using a polymer adds a polymer layer around the Pd-AGS and can retard diffusion and transfer of substances between the AGS and aqueous phase. In addition, the active sites of the Pd catalyst may be covered by the polymer or fouled by other organic or inorganic species during the immobilization process. All these factors may contribute to loss of catalytic activity after polymer immobilization. Therefore, polymer immobilization is not suggested as a stabilization strategy for Pd-AGS during application.

## Conclusion

Biogenic PdNPs were *in situ* formed by AGS, and the Pd-AGS was for the first time used for iohexol degradation. The Pd-AGS demonstrated a much higher iohexol reduction rate than the AGS control, and had similar reactivity to free Pd powder. A nearly complete removal of iohexol (20 mg/L) was achieved using the Pd-AGS within 2 h in the presence of formate as the electron donor. As a combination of a Pd catalyst with a live microbial culture, a variety of organic compounds could serve as the electron donor to initiate the catalytic activity of Pd-AGS. The efficiency of the tested electron donors was in the following order: formate > lactate > ethanol > glucose > acetate. The removal of iohexol followed the Langmuir–Hinshelwood model and the conversion of adsorbed iohexol on the surface of Pd-AGS was the rate controlling step. The Pd-AGS had high reactivity in DI water at mild pH, and almost no reactivity in acidic (pH = 1.2) and alkaline (pH > 11) media. The presence of iodide ion in the medium also reduced the Pd-AGS catalytic activity. Polymer immobilization is not suggested for the Pd-AGS as it may greatly decrease the reactivity. The Pd-AGS provides a novel strategy for iohexol removal from slightly polluted water.

## Author Contributions

XQ designed the experiments and wrote the manuscript. XZ and YS performed the experiments and data analysis. JZ provided analytical support.

## Conflict of Interest Statement

The authors declare that the research was conducted in the absence of any commercial or financial relationships that could be construed as a potential conflict of interest.
