# Supplementary material for: Iohexol Degradation by Biogenic Palladium Nanoparticles Hosted in Anaerobic Granular Sludge
Source: Front Microbiol. 2018 Aug 23;9:1980. doi: 10.3389/fmicb.2018.01980 (PMC6115513; doi:10.3389/fmicb.2018.01980)
Supplement: Supplementary file 1 [file Data_Sheet_1.PDF]

Supplementary Material

Iohexol degradation by biogenic palladium nanoparticles hosted in anaerobic granular sludge

Xiangchun Quan*, Xin Zhang, Yue Sun, Jinbo Zhao

Key Laboratory of Water and Sediment Sciences of Ministry of Education, State Key Laboratory of Water Environment Simulation, School of Environment, Beijing Normal University, Beijing 100875, China

*** Correspondence:**
Corresponding Author
E-mail: xchquan@bnu.edu.cn

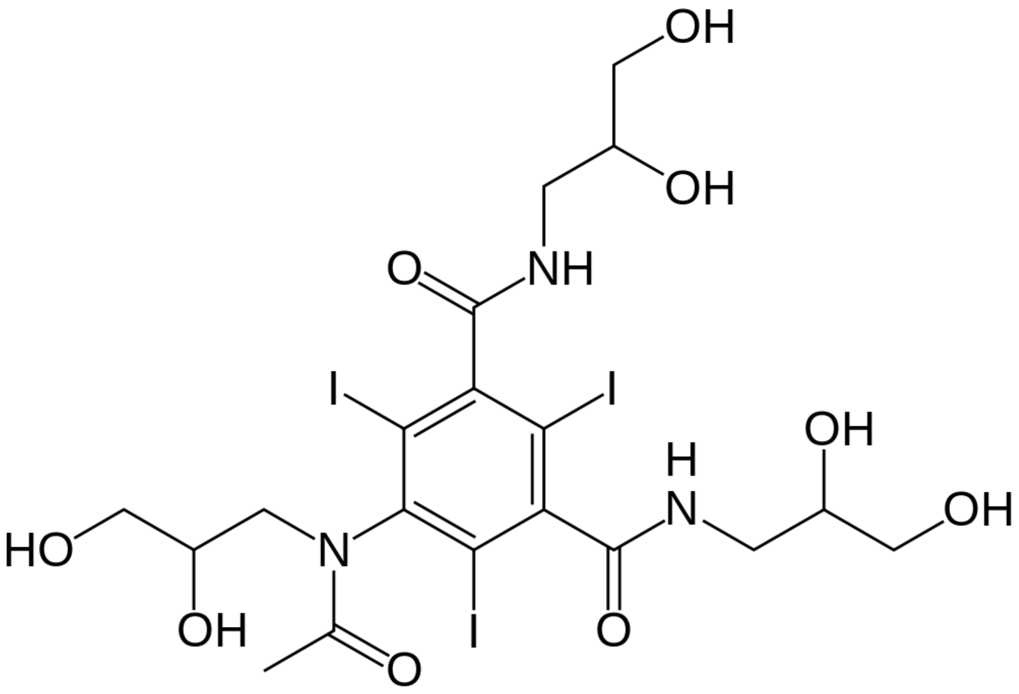

Figure S1. Chemical structure of iohexol

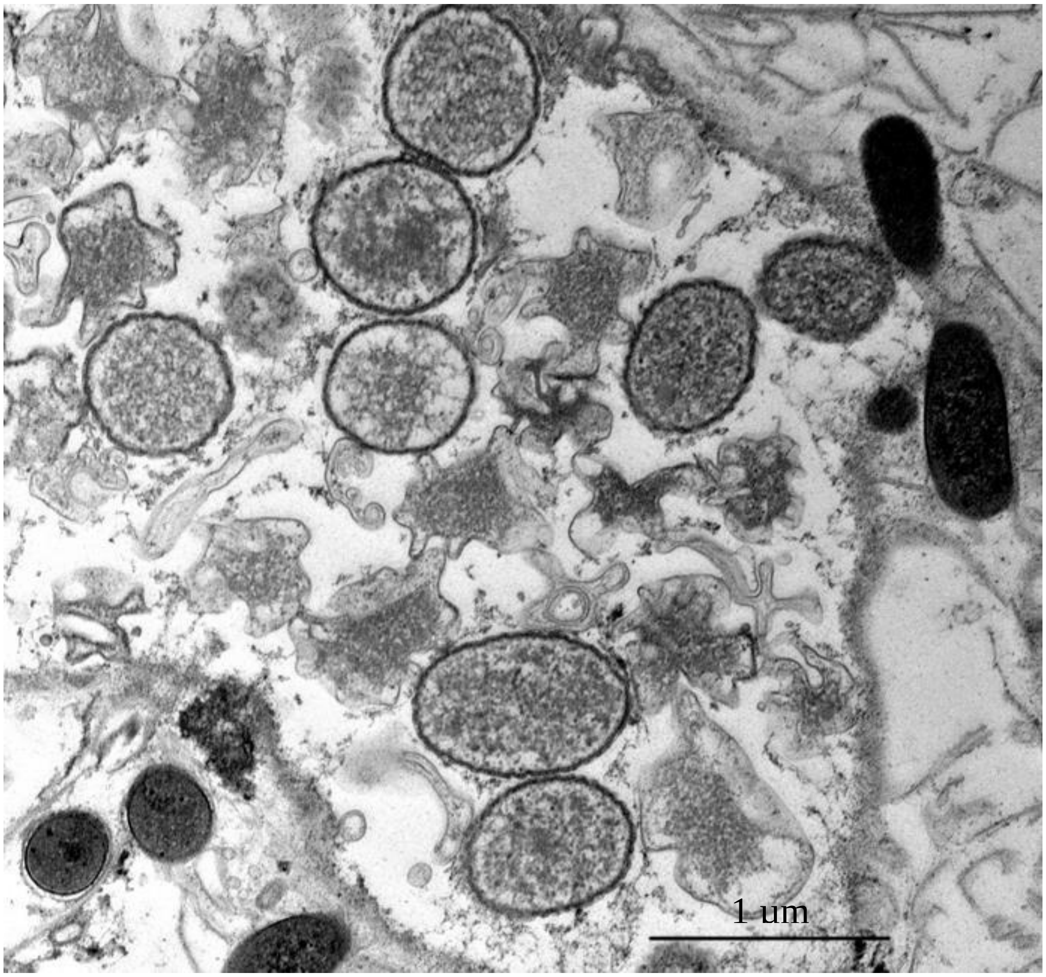

Figure S2 TEM images showing the presence of PdNPs in the microbes of AGS
